# A complex polymorphous psychosis or a cycloid psychosis with a different onset?

**DOI:** 10.1192/j.eurpsy.2023.2246

**Published:** 2023-07-19

**Authors:** H. Becerra Darriba

**Affiliations:** Psychiatry, Osasunbidea, Tudela, Spain

## Abstract

**Introduction:**

Acute and transitory psychotic disorders comprise a polymorphous picture such as Leonhard’s cycloid psychoses, which alternate episodes of affective symptoms such as psychosis between two poles (anguish/happiness, incoherence/stupor, or akinesia/hyperkinesia).

**Objectives:**

To describe a case report of a 20-year-old man, in outpatient psychiatric follow-up, after debuting at age 18 with a severe depressive episode of endogenomorphic characteristics without psychotic symptoms, with subsequent complete remission. Two years after clinical stability, he required prolonged hospitalization due to polymorphous psychotic syndrome of abrupt onset in a context of previous continuous use of cannabis and cocaine. Suspicion towards parents, bizarre behaviors, rushing desires, unmotivated laughter, fixed gaze, bewilderment, anguish with a feeling of imminent death, alternates with euphoria and senseless purchases.

**Methods:**

We present the case report of this patient with a mental examination of conscious, scattered attention with marked distractibility, confusion and experiences of strangeness, memory gaps, subjective sensation of well-being with tachypsychia, which fluctuates with thymic oscillations and alternates with episodes of marked indefinite anguish, intense anxiety with delusional fear of the death of him or his family. Little systematized ideas of reference and prejudice based on intuitions or delusional occurrences in their environment. Megalomaniac and religious-messianic ideation. No sensory perception disturbances. Disintegrated course of thought, with frequent illogical associations, ambivalence of thought, affectivity and psychomotricity. Motor restlessness and behavioral disorganization. Global insomnia. Judgment of reality and superior functions diminished. No auto/heteroaggressiveness.

**Results:**

Various psychoactive drugs were tested for two months, obtaining a response only with valproic acid 1500mg, pregabalin 450mg and olanzapine 15mg, presenting slow improvement in a situation of absence of consumption, with a predominance of symptomatic polymorphism, decreasing fluctuation between episodes of expansiveness and psychotic anguish, remitting disorganization and motility alterations, persisting poor awareness of the disease and cognitive complaints. He was referred for follow-up at the mental health center where his gradual recovery continued.

A differential diagnosis of polymorphous psychosis is proposed, compatible with a cycloid psychosis of the anxiety-happiness type with marked affective symptoms, precipitated by substance use.

**Conclusions:**

Cycloid anxiety-happiness psychosis stands out for intense and fluctuating anxiety, oscillating with feelings of happiness, ecstasy, and placidity, mystical-religious delusions, and preoccupation with death, which may comprise a different psychotic debut.

**Disclosure of Interest:**

None Declared

